# How do Pregnant Women with Additional Health or Social Care Needs Experience Parenting Groups: Evidence from Delivery of Enhanced Triple P for Baby and Mellow Bumps as Part of the Trial of Healthy Relationships Initiatives in the Very Early Years (THRIVE)

**DOI:** 10.1080/13575279.2021.1933902

**Published:** 2021-06-17

**Authors:** Katie Buston, Alice MacLachlan, Marion Henderson

**Affiliations:** a MRC/CSO Social and Public Health Sciences Unit, University of Glasgow, Glasgow, UK; b School of Social Work and Social Policy, University of Strathclyde, Glasgow, UK

## Abstract

There is still relatively little known about when, why, how and in what circumstances parenting interventions are effective. Support within the group context has been theorised as a key mechanism. This paper explores how pregnant women with additional health or social care needs participating in two group parenting interventions—Mellow Bumps or Enhanced Triple P for Babies—experienced being in a parenting group, and how this shaped how they engaged with the interventions; and it examines how group delivery may have facilitated or inhibited the effectiveness of the interventions, and for whom it did so. Session evaluation forms (*n* = 708) and a post-intervention questionnaire (*n* = 117) were completed by participants. In-depth interviews were conducted following the MB/ETPB antenatal sessions (*n* = 19), and 6–12 months after the birth of their baby (*n* = 15). Group delivery of these parenting interventions had the potential to support participants, particularly those with multiple additional health and social care needs. There are, however, important caveats including patchy attendance reducing the supportiveness of the groups, and few discernible longer terms changes. More group sessions, less patchy attendance, and more encouragement from facilitators for the women to keep in touch, and to join other community parent–child groups after the birth of their baby are likely to have increased feelings of support and connectedness.

## Introduction

Parenting programmes have been shown to be effective in supporting parents to develop the behaviours they need in order to meet the needs of their child(ren). When, why, how and in what circumstances they work is, however, less clear (Datta & Petticrew, 2013; Kane et al., 2007). Identifying “active ingredients” underpinning effectiveness is challenging (Craig et al., 2008; Wells et al., 2012); it is helpful to elucidate mechanisms of action behind the potential behavioural change in order to answer these questions.

The theory of change for group-based interventions commonly points to the importance of support within the group context as a key mechanism of action, helping us understand how behavioural change might occur. In a systematic review and synthesis of qualitative research on parenting programmes, Kane et al. (2007) point to the importance of feelings of acceptance and support from others in the parenting group. This contributed to parents regaining control and feeling more able to cope, and to reductions in feelings of guilt and social isolation, increased empathy with their children, and greater confidence in dealing with their behaviour. A systematic review of randomised controlled trials that compared group-based parenting programmes with a control condition found that the types of support derived from group programmes improved short and medium-term parental stress and confidence (Barlow et al., 2014). Buston (2018) points to the development of positive relationships between participants in the group and between participants and the programme facilitators as creating a supportive atmosphere, which is conducive to engagement, active participation, and, therefore, to enabling change as intended in the programme’s theory of change. This supportive environment may be particularly important for those who are living under stress and have additional needs (Sheffield Morris et al., 2017). Erickson et al. (1992) found that having mothers together in a group helped them examine painful childhood experiences and feelings because they felt less alone listening to the similar experiences of other mothers. Polansky et al. (2006) found that for parenting groups for high-risk mothers living in a residential programme, being understood and accepted by other group members in a supportive and non-judgmental environment was particularly important (see also Schelbe et al., 2018), facilitating reflection within the group and thus bolstering the aims of the programme. All of these pathways can, potentially, lead to positive change (Balaji et al., 2007).

Here we focus on how pregnant women with additional health or social care needs participating in two group parenting interventions—Mellow Bumps (MB) (Mellow Programmes, 2020) and Enhanced Triple P for Babies (ETPB) (Triple P, 2020)—experienced being in a parenting group and how this shaped how they engaged with the interventions. MB is underpinned by attachment theory, designed to target mothers who are vulnerable in pregnancy. ETPB incorporates social learning principles with an emphasis on families. Theories of change for each intervention have been published (O’Brien et al., 2019). Both interventions were delivered by two trained facilitators, to groups of up to eight women, from 20 weeks of pregnancy. By examining the experiences of the women participating in these programmes, and their engagement, we can better understand the potential effectiveness of these and other similar group-based interventions, and some of the mechanisms which may lead to behavioural and other change.

The Trial for Healthy Relationships Initiatives in the Very Early Years (THRIVE) is a randomised controlled trial (RCT) that evaluated ETPB and MB for pregnant women with additional social and health care needs (Henderson et al., 2019; O’Brien et al., 2019). Here we present and reflect on participant experiences of, and engagement with, the group from the perspectives of the participants themselves. We explore the ways in which this group delivery mode may have facilitated or inhibited the effectiveness of the two interventions, how it did so, and for whom (see Shearn et al., 2017). Specifically, we explore: how the women felt about being in the groups, and what kind of support they offered; how conducive the group atmosphere was to active participation and how listening to others shaped engagement; reported change as a result of participating in the groups; and how the women’s vulnerabilities shaped their engagement. Findings will inform understandings of how group-based parenting interventions are experienced, particularly by women with additional health or social care needs, and how, when, and for whom they may be effective.

## Interventions and methods

### Study design and population

THRIVE was a three-arm RCT that evaluated the impact of two parenting interventions—MB and ETPB—both with care-as-usual (CAU) versus CAU alone, on maternal mental health and mother-child interactions in women with additional health and social care needs in pregnancy (trial registration: ISRCTN21656568) (Henderson et al., 2019). It comprised an inter-linked Outcome Evaluation, Economic Evaluation and Process Evaluation (PE). This paper draws mainly on the PE. Ethical approval for the study was granted by NHS West of Scotland Research Ethics Committee (13/WS/0163, 21 August 2013). Informed written consent was obtained from all participants prior to all data collection and participation in the intervention.

**Figure 1:**
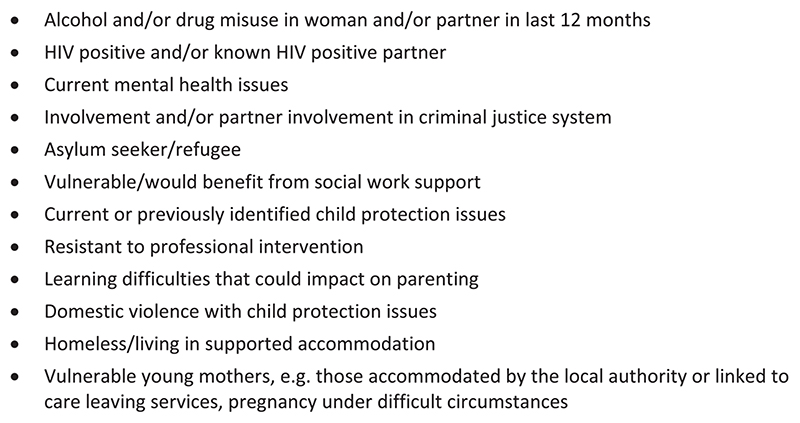
NHS greater Glasgow & Clyde special needs in pregnancy criteria.

**Figure 2:**
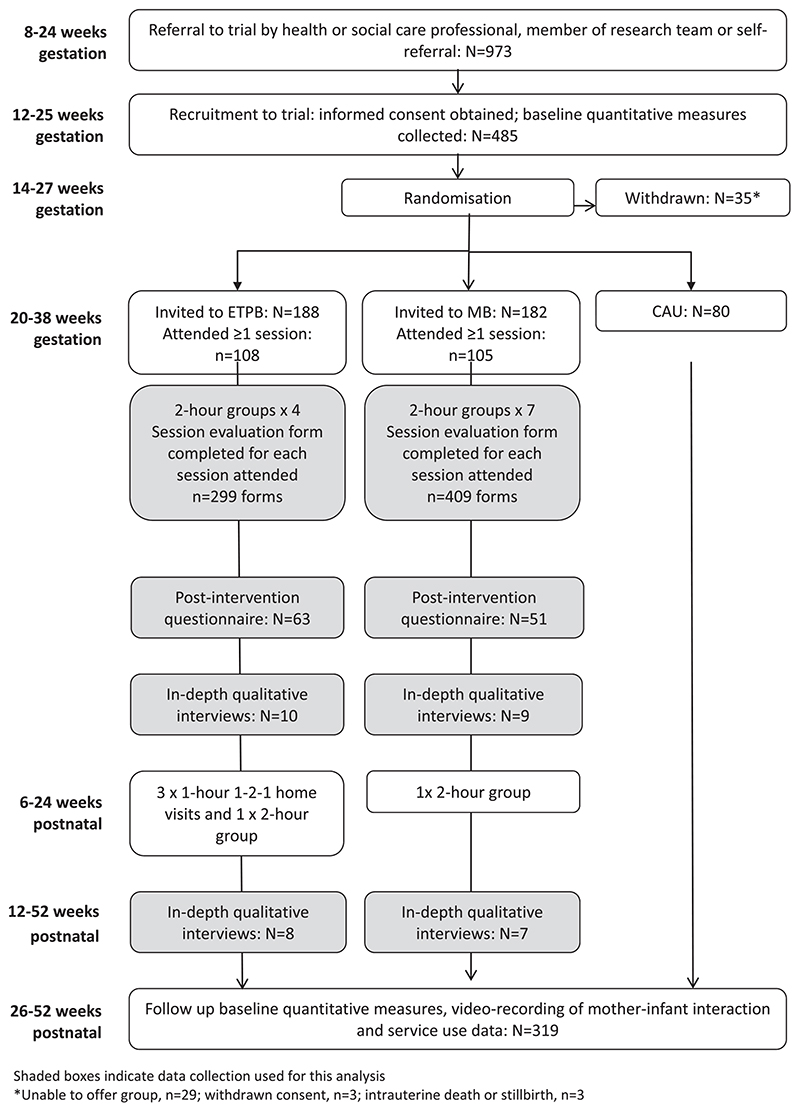
Study flowchart.

Pregnant women eligible for the trial were identified via referral from a health or social care practitioner or a member of the study team or by self-referral. Women eligible to participate in THRIVE were aged 16 or older (or 14 or older with social work support) living within the NHS Greater Glasgow and Clyde and NHS Ayrshire and Arran health boards in Scotland who met one or more of the NHS Greater Glasgow and Clyde Special Needs in Pregnancy criteria (see Figure 1) (Glasgow Child Protection Committee, 2008). A referral form was completed for each participant, which included details of the additional health and social care needs making them eligible for the study. Further information on these needs was collated through responses to the baseline study questionnaire and ethnographic observations. In total, 485 women were recruited to the trial. Of these, 182 were invited to attend MB sessions and 188 invited to attend ETPB sessions. Two participants were under 16 at randomisation; the average age of the sample was 26.7 (standard deviation 6.3). Overall, 319 women completed all data follow-up, of whom 131 had been invited to MB sessions, and 135 to ETPB sessions. The study flowchart (see Figure 2) provides more detail (see also Henderson et al., 2019; Henderson et al., forthcoming; O’Brien et al., 2019).

### The interventions

The MB programme includes six core antenatal group sessions for mothers-to-be, an optional antenatal session including partners, and a postnatal group reunion session. Nurturing, relaxation and support of participants and the facilitators are theorised as a central mechanism of change leading to greater mother-infant attachment (Puckering et al., 2010). The social aspect of the group delivery underpins the intervention aims of improving reflective functioning and parenting skills. The ETPB programme includes four antenatal group sessions, postnatal one-to-one sessions, and a further postnatal group session; women are encouraged to bring a partner or other support to all sessions. For MB, the “closed” and “safe” group is key to its theory of change, whereas for ETPB the group aspect is less core but there is recognition that discussion about parenting attitudes and behaviour and the practising of skills within the group are important (Prinz et al., 2009).

Given the differences in delivery of the postnatal sessions (one group session for MB; a mix of one-to-one and a group session for ETPB) this analysis focuses on the group antenatal sessions only. A brief summary of the content of each antenatal session, by intervention, is provided in Tables 1 and 2 (see also O’Brien et al. (2019) and Buston et al. (2019)).

All women participating in THRIVE continued to receive their routine antenatal and postnatal care (CAU) provided through the UK National Health Service (NHS). Women were permitted to take up other parenting programmes outside of THRIVE if they chose to. This uptake was captured through a service use diary.

### Data collection and analysis

As part of the PE, session evaluation forms were distributed to all the women participating. They were collected following each session (MB: *n* = 409 forms; ETPB: *n* = 299 forms) in order to glean information across the sample on every session. The response rate was 87% for MB (75-100% across individual sessions) and 93% for ETPB (85-97%). In addition, a post-intervention questionnaire was completed after the last antenatal session for each group (MB: *n* = 51; ETPB: *n* = 63). This questionnaire asked women to reflect on their experiences across all of the sessions and if/how the group had helped them to prepare for motherhood. Of the women who had attended at least one intervention session, 46% completed the post-intervention questionnaire for MB and 59% for ETPB. The session evaluation forms and post-intervention questionnaires included a series of statements with Likert Scale responses (e.g. “I felt listened to”, 5-point Likert Scale from Strongly Agree to Strongly Disagree), and questions for women to provide free text comments (e.g. “What did you like about today’s session?”). The majority of free text comments were 1–2 brief sentences. Descriptive summary statistics are provided for Likert Scale responses. Free text comments were reviewed for each session, by AM, and those related to the group context were identified and categorised into broad themes.

To supplement the breadth provided by the forms and to provide richer data on views and experiences, in-depth interviews were conducted with 19 women who participated in MB (*n* = 9) or ETPB (*n* = 10) following completion of the MB/ETPB antenatal sessions, and before the birth of the baby. The women were purposively sampled to provide heteorgenity with regard to: age, NHS region, attendance (at 50% or more of intervention sessions), parity and vulnerability. The interviews focused on: the background of the women, including the nature of their additional social and health care needs; the circumstances surrounding their pregnancies; their experiences of being a parent and of being parented; their experiences of recruitment to the trial; their understanding of and response to key mechanisms theorised for the interventions; and their thoughts, on and experiences of, participating in the interventions. Further in-depth interviews were conducted with fifteen women who participated in MB (*n* = 7) or ETPB (*n* = 8) (six of the women took part in the earlier interviews also), six months to a year after the birth of their baby. They focused on: how their lives had been since their babies were born and whether or not there had been any sustained benefits, or negative effects, of participating in the interventions. The number of interviews conducted allowed for exploration of heterogeneity across the women and their experiences, while being within the capacity of the trial staffing given the time-consuming nature of organisation, data collection and analysis.

The interviews, and accompanying fieldnotes, were analysed using the framework approach (Gale et al., 2013), as part of the PE (see O’Brien et al., 2019 for further details). First level descriptive and thematic coding was conducted as part of efforts to understand how the interventions were being delivered and received. KB then focused on analysing the data on the group mode of delivery. This involved more explanatory analyses, using the descriptive codes as a starting point and interrogating the data to better understand how and why the women experienced the groups in the way they did, and for whom group delivery may have been beneficial. The initial coding frame for these analyses was formulated by drawing on relevant literature on mechanisms of action deriving from group delivery, primarily for parenting interventions (e.g. Kane et al., 2007; Polansky et al., 2006). Codes included: feeling accepted, feeling less alone, the importance of feeling comfortable during intervention delivery. Particular attention was paid to how the women related their feelings about being part of the group to their discourse on how, if at all, the interventions had made a difference to parenting and other outcomes, and to the women’s additional health and social care needs, and how these might be shaping their experiences and discourse. Deviant case analysis was used to refine the process (Pope et al., 2000). Triangulating across THRIVE data sources allowed analysis by the delivery group in order that the characteristics of women within each group and group dynamics of individual groups could be incorporated.

## Results

The research questions explored within this analysis are: (i) how the women experienced the groups, including how they felt about being a part of the groups, and what kind of support the groups offered; (ii) how engagement with the intervention was shaped by the group delivery context, including how conducive the atmosphere was to active participation and how listening to others shaped engagement; (iii) what changes the women reported as a result of being a part of the groups, and (iv) how the women’s vulnerabilities shaped their experiences. First, we present data on group delivery and attendance.

Twenty-eight MB groups and 30 ETPB groups were delivered. In total, 182 women were invited to attend an MB group, 108 (60%) attended at least one antenatal session. Of these women 78% attended at least half of the antenatal sessions, 19% attended all six core antenatal sessions. The optional partner session was run for three groups; within these groups, two women attended all seven of the sessions. In total, 188 women were invited to attend ETPB, 105 (56%) attended at least one session. Of these women, 84% attended at least half of the sessions, 52% attended all four antenatal group sessions. Of the 101 women for whom we have data on partner attendance, 58 (57%) attended at least one session with a partner or other support person (e.g. friend). Between 2 and 12 women were invited to attend each group: group sizes ranged from 2 to 6 women for MB and 1–7 women for ETPB. For MB the number of women attending the first session ranged from 1 to 6 and for ETPB 1–5. Across groups, the average number of women attending a session was 2.7, for both interventions. Only a small proportion of women attended all the group sessions. For many others attendance was inconsistent rather than there being a sustained drop-out over time. Data collected about reasons for non-attendance indicated that this was not usually related to the programme itself, but rather other issues such as sickness during pregnancy, not being able to get time off work, or feeling too anxious to attend.

### (i) How the women experienced the groups: how they felt about being a part of the groups and what kind of support the groups offered?

Based on responses to the post-intervention questionnaire (MB, *n* = 51; ETPB, *n* = 63), almost all of women found the groups supportive; MB: 71% very supportive, 27% quite or moderately supportive; ETPB 65% very supportive, 33% quite or moderately supportive. In terms of relationships with other women in the group, for MB 31% rated their relationship as excellent, 33% as very good, 29% as good, 6% as fair, and none as poor, while for ETPB 27% rated their relationship as excellent, 29% as very good, 35% as good, 8% as fair, and 2% as poor. There was no statistically significant difference in responses between the interventions (Mann-Whitney, *p* > .05). When asked about friendships formed with others in the group, among MB participants the mean number of new friendships formed was 2.1, although 22% stated that they did not become friends with any women in the group. For ETPB the mean number of new friendships was 1.1, with 35% stating that they did not become friends with any women in the group.

For MB many of the written comments for each individual session evaluation highlighted aspects of the group the women had found positive including: meeting new people; hearing about and sharing experiences and opinions with other mothers-to-be; a friendly, supportive, informal, welcoming and comforting group atmosphere; having the opportunity to speak openly, and talk about concerns; feeling listened to; forming intimate bonds with others in the group and small group size. A small number of comments highlighted negative aspects: group too small; awkward conversations; over-talking from some dominant individuals; and not enough opportunities to communicate with group members.

For ETPB the written comments for each session highlighted positive aspects of the group including: the opportunity to be involved in group discussions; meeting other mums; being able to listen to, relate to and share experiences with others; being able to talk about their own experiences and feelings; friendly participants; and small group size. A small number of comments highlighted negative aspects: group too small; negative impact of other individuals within the group on the group dynamic.

The interviews elucidated ways in which the groups were supportive. For MB participants, the aspect identified by most women was the reduction in isolation that the groups offered. Eight women talked about the positive aspects of getting out of the house to attend the group and interact with other participants:

it’s good to have people about to talk to and things like that, because you do, you feel lonely when you’re in the house not being able to do anything. If you’re out and about with other people then, yeah, it’s great. You don’t feel as down and depressed. (Nelly, MB, mental health difficulties)

The opportunity to mix with, chat to, and moan alongside other women who were at the same stage of pregnancy was identified as important by many of the MB participants:

I think that was what was really good about Mellow Bumps as well was having a sense of humour and just being able to kind of talk about stuff and laugh about stuff, and then reflect …. Just having that space to talk openly … just to be able to talk about how you feel and stuff like that. (Rita, MB, mental health difficulties, past homelessness)

Indeed, a small number of those interviewed pointed to the therapeutic nature of sharing their experiences, going beyond talk of pregnancy health issues and niceties to deeper issues, such as their backgrounds and current living situations:

it [the group] was actually very therapeutic for me. Very. I just felt just relieved every time I came home. Like, on the way home I thought ‘wow, I’ve spoke about that, and I’ve got that out’. (Jane, MB, past substance misuse, violent relationship, mental health difficulties, homelessness, maltreated as child)

it really helped me. Before that I was thinking maybe I’m the only person having these things, you know. When I claim asylum then everything, when they moved me to [name of town] … I met with these ladies and I felt that I’m not alone in the world having these problems. (Kala, MB violent relationship, mental health difficulties, homelessness, child-hood abuse)

There were far fewer negatives than positives expressed about the groups and the support they offered. Two of the women interviewed complained about having nothing in common with other group members, and another described conflict with another group member which limited the support she felt the group offered. These women did, however, enjoy the group experience. Only one woman talked about finding the group experience negative, and distressing:

I was looking forward to it and that’s why I got my midwife to refer me, and even though I went every week … I didn’t feel comfy, felt as if I wasn’t involved … It’s not a nice feeling to feel like that especially when you’re there for the same reason as them. (Isla, MB, substance misuse, partner substance misuse, violent relationship, mental health difficulties, looked after as a child, homelessness, child protection concerns - social wok involvement, maltreated as child)

Overall, however, the women described finding the MB groups positive and supportive for as long as they were running.

For ETPB, the picture was similar in terms of positive assessments of supportiveness, but none of the women talked about the groups being therapeutic. Rather, the support came from sharing relatively superficial experiences and issues relating to being pregnant and becoming a parent, without much reference to their own backgrounds/situations, and without a sense of “unburdening”one’s deep-seated problems in the groups. The presence of some of the women’s partners in the groups also contributed to differences in the way the women attending ETPB talked about group support. This added an additional, dimension of support for most of the women who attended with their partners:

we learned so much from it and I think it helped to bring us closer together as well because we could see both sides and everybody else’s as well. You’re sitting listening to other people, you realise you’re not quite as alone as what you think. (Annie, ETPB, past substance misuse, violent relationship, mental health difficulties, family history mental health difficulties, past homelessness)

There were, however, more similarities than differences between the MB and ETPB participants in terms of how they talked about group support. Being able to form enduring bonds and friendships was something many of the women mentioned, for example, as something they had hoped to get out of their attendance. However, only a very small number of those interviewed formed these sorts of ties. The short duration, in weeks, of the programmes, small size of the initial groups, and often low and patchy attendance beyond this, were regarded as barriers to forming connections within and beyond the groups. The greater number of MB group sessions as compared with ETPB may have made a small difference as the MB participants felt slightly more connected to others in the group. Partner attendance at ETPB may also have contributed, as might the more in-depth nature of discussions at the MB sessions. However, the patchy attendance for both programmes appeared to be more important in impeding the formation of longer-lasting bonds. There was also a perception amongst some of the MB participants that the facilitators had not encouraged keeping in touch. Therefore, while enjoying the chat and support within the sessions, most of the women attending the interventions said they did not feel a sustainable connection with other participants:

I think I bonded in so much that we could bond. Like, because I think when you’re pregnant it’s, kind of, and we live so far away from each other really, it’s kind of difficult to give any, kind of, friendship commitment beyond what you’ve already got. But I think we bonded within the group. Not outwith it (Meg, ETPB, past substance misuse, partner substance misuse, mental health difficulties)

A small number of women reported, at the time 2 interviews, having formed longer-lasting ties. These women saw this as a very positive outcome of group attendance, having formed enduring friendships with women with children of similar age:

the three of us all were in there [group] with similar mental health issues as well so we had a lot in common…we message each other like “oh this is going on” and we all reassure each other, you know “yeah, that’s happened to me” or “yeah that’s normal”. (Zoe, ETPB, violent relationship, mental health difficulties, maltreated as a child)

### (ii) how engagement with the intervention was shaped by the group delivery context

Based on the 409 session evaluation forms collected across the antenatal MB sessions, almost all of those attending agreed or strongly agreed that they felt they could speak when they wanted to (95–100% across sessions) and that they were listened to (91–100% across sessions). Seven of the 108 MB participants reported feeling left out of a session. Four stopped attending. Similarly for ETPB, the 299 session evaluation forms collected across the antenatal sessions showed that almost all participants agreed or strongly agreed that they felt they could speak when they wanted to (97–99% across sessions), and were listened to (97–100%). Seven of the 105 ETPB participants reported feeling left out of a session, though they continued to attend.

There was variation in the women’s reported engagement with the groups, but this variation could not be attributed to whether they participated in MB or ETPB. Nearly all said they had enjoyed the groups and found them worthwhile. Most talked about turning up, participating in, and getting something out of the sessions. A smaller number were more profuse in their feedback, talking about how important the sessions had been to them, and about how they had engaged fully in the groups.

Nearly all of the women reported that the facilitators were successful in creating an atmosphere within the groups that facilitated comfort, engagement and participation. They also said that the facilitators offered concrete support via sharing their own experiences:

what I do think helped was that the coordinators of the course opened up about their own personal life experience of things…It was good because it didn’t feel like an us and them. (Rita, MB, mental health difficulties, past homelessness)

Most of the women talked about the atmosphere within the groups being conducive to supporting each other. Most groups were small on any given week; some of the women commented that they wished there had been more participants, others that they liked that the groups were small. Some felt a bigger group made participation and sharing more worthwhile, while others found it easier to contribute in smaller groups. What was seen as more important, though, was consistent attendance. Consistent attendance was valued, different attendees each week could change the group dynamic and disrupt bonding, making the atmosphere less relaxed and supportive.

### (iii) what changes do the women report as a result of being a part of the groups

When asked how attending the groups had helped them, 84% of those who completed the post-intervention questionnaire for MB agreed or strongly agreed that the group had made them more confident talking to others; 84% that it had made them more confident about being a mother; 82% that it had helped them feel better about themselves; 78% that it had helped them become more aware of how they were feeling; 76% that it had helped them accept themselves; and 67% that the group had made them less anxious about being a mother (Table 3).

Among those who completed the post-intervention questionnaire for ETPB, 94% agreed or strongly agreed that the group had made them more confident about being a mother; 87% that it had made them feel better about themselves; 86% that it had made them less anxious about being a mother; 79% that it had helped them accept themselves; 77% that it had helped them become aware of how they were feeling; and 73% felt more confident talking to others (Table 3).

Only a small number of the women talked about changes as a result of attending the groups during the time 2 interviews, 6-12 months after the birth of their baby.

Across both programmes, a small number of women talked about the groups being helpful in making them realise it was fine to be anxious and unconfident about parenting, engendering a sense of enduring relief and legitimating their emotions:

what is really good is when you realise that everyone is not necessarily the same but everyone has fears. Cos often people think “oh, everyone else is coping so well, everyone else is doing so well”, and once you sit down and started talking about things then you realise everyone’s just as anxious …. It’s starting opening up dialogue for people, saying it’s okay to not be okay. (Rita, MB, mental health difficulties, past homelessness)

A small number of women also talked about the groups facilitating long-lasting supportiveness::

See from this as well, I’m thinking now I would want to go to like Mothers and Toddlers …. Because I think just going with a group of women and sit and having a wee chat … it’s kinda made me think about doing all these wee things that I probably wouldn’t have thought about before. That’s just what made me more confident. (Alex, MB, substance misuse, violent relationship, mental health issues)

### (iv) how the women’s vulnerabilities shaped their experiences

Many of the women, particularly those with multiple vulnerabilities, reflected on how their own backgrounds and experiences had shaped their group experience.

Anxiety was often referred to, potentially hampering the women’s first visit to the groups. Other aspects of their lives and backgrounds were also referenced. For Kala, for example, the groups were a welcome (and rare) opportunity for her to talk about her situation as an asylum seeker who had experienced domestic abuse and was now living alone, and to hear that other women in her group had gone through similar experiences:

I know for the people to share the things is very difficult. And Mellow Bumps, yes, because you met other people who suffered the same things. When they shared then you have the courage, then you get some courage. Yes, she shared so I should share as well. And they make it feel like that, you know that people can share their things, they can take something from their inside and put it out because when things come outside I think you will feel relaxed … because some time when I thought all those things it all comes in my mind, when I don’t have anybody to talk to then I cried. I cried and then, you know, some time I harm myself… for the lonely person especially it is really good to share all these things. (Kala, MB, violent relationship, mental health difficulties, homelessness, childhood abuse)

The groups were appreciated most by the women with multiple vulnerabilities, many of whom valued sharing their situations and hearing about those of the other women. This was particularly so for those attending MB.

The exception was Isla (see above) who felt left out of the MB group she participated in. However, she continued to attend, perhaps because she had already had multiple children removed from her care, and believed that if she kept attending she would increase her chances of being able to keep this baby. Isla was described by another member of the group as “looking different” and her habit of leaving the group several times during each session to smoke a cigarette was commented upon disapprovingly. Isla felt she was not being invited to share her experiences in the same way that the other women were. As such, she felt constrained from participating fully:

I didn’t tell them [other women in the group] that my kids were in foster care … I’d told [name of facilitator] when she came out like to see me … But then like when it was questions about kids I felt as if they made it obvious “Oh, her kids aren’t with her”, cause it was always aimed at the others, they never asked me one question about my kids and that, and then it was like the other women would be sat and they’d be like looking as if to say “why are you asking us but not her?” and then every time it come round to kids [name of facilitator] would turn round and she’d go “but everybody’s circumstances aren’t the same”, but that, to me, felt as if it were just like a hint towards “oh, her kids aren’t with her”…It might just have been me but I felt as if I didn’t fit in with them. (Isla, MB, substance misuse, partner substance misuse, violent relationship, mental health difficulties, looked after as a child, homelessness, child protection concerns - social wok involvement, maltreated as child)

It is plausible that Isla would not have felt this if she had been in an ETPB group, due to MB delving more deeply into participants’ backgrounds during reflective work.

## Discussion

It can be challenging to distinguish intervention content and delivery context when exploring the effectiveness of parenting interventions. Many parenting interventions are delivered in group settings, with the support provided through the group context critical to behavioural change. By comparing two group-based parenting interventions delivered and evaluated within the same RCT, we have been able to focus on the specific contribution of the group context to the experiences of participants. The key question was: how did the women feel about being part of the MB and ETPB groups, and what kinds of support did the groups offer?

The group delivery context was successful. The majority of the women liked attending the groups. For such “hard to reach” women the high levels of attendance and satisfaction with the groups might seem surprising (Evangelou et al., 2013). Support gleaned from the groups was highlighted by most participants. Being a part of something with other women at the same stage of pregnancy, sharing experiences and thoughts with these women, and, for some of those attending MB, being able to do this therapeutically was valued highly. Having the programmes delivered in groups certainly seemed to contribute to these women attending, and to their positive assessment of their experience of the interventions, boosting their engagement overall, and with the sessions that they attended. The groups allowed the women to move beyond didactic learning and engage by contributing to the group, and hearing others contribute to it. Whether attitudinal and/or behavioural changes occurred as a result, however, is less certain. The women’s enjoyment of the groups, and their positive assessment of what they got out of them and the intervention overall, seemed to be situated more in the moment of the programmes running, and for a short period thereafter. Among the 15 women interviewed more than six months after completing the intervention, few enduring changes were identified. What did seem to be a long-lasting benefit for a small number was lasting friendships formed with others in the group. Confidence to attend post-natal groups, such as mother and toddler groups, where continued support could be gleaned, was valued. Many of the women participating in THRIVE have multiple, and often deep-seated, vulnerabilities. Generally, those with a greater number of additional health and social care needs reported getting the most from the group aspect of delivery. There was only one woman who reported a detrimental, and possibly harmful, group experience. Her experience should be considered carefully. Her perception that she was excluded because of her past and current addictions, and the removal of previous children, is important. She had continued difficulties in her life as she was about to give birth again; a negative experience of the intervention is a harm that should be given weight. Generally, however, the groups seemed to be particularly supportive for these very vulnerable women.

Group delivery of social interventions, such as the parenting interventions focused on here, appear to have the potential to support participants, particularly those with complex needs (Kawachi & Berkman, 2001). There are, however, important caveats. First, within the “real world” implementation of these groups, patchy attendance reduced their supportiveness. Consistent attendance and the formation of a coherent group was important. The women felt stable membership made for a more supportive group than one where the membership differed from week to week and dynamics changed. It was harder to develop rapport when attendance was patchy (Buston et al., 2019). When considering the implementation and delivery of parenting interventions more generally, it is unsurprising that in the often busy, and sometimes chaotic, lives of pregnant participants who may already have other children, or were facing difficulties, consistent attendance was not always viable. Efforts to retain participants and to facilitate their consistent attendance should be a core part of implementation plans, though this is challenging (see also Robinson et al., 2018). It requires a clear understanding of the nature of participants’ lives at the design stage. For THRIVE, taxis were organised, and reminder phone calls were made to the women, yet attendance was still patchy. Second, support was, generally, felt only for the short time the groups were running, with few discernible longer-term changes or benefits emanating from it.

A strength of this study is its focus on vulnerable mothers/mother-to-be. Parenting, and other, interventions may be of particular importance in supporting this population. What conclusions can we draw from this, pertinent to this particular population? Polansky et al.’s (2006) smaller-scale study of mothers with drug addictions attending a 6-week group-based parenting programme found that being understood and accepted as part of a group gave them a profound sense of security (see also Robinson et al., 2018). This was of particular value to these mothers who had had difficult family lives as children, and into adulthood, in common with many of those taking part in THRIVE. Shared histories can be potent here (see Lindgren, 2014). Social support for mothers by mothers is also powerful, especially when this might be lacking elsewhere (Erickson et al., 1992). Generally, those women we interviewed who appeared to have faced the most difficult situations in childhood and/or into adulthood were the ones who valued the groups the most (Robinson et al., 2018). It is plausible that the groups were providing them with the support and affirmation that they were not able to access elsewhere, for example from a supportive partner. Part of the strength of this was in finding themselves in groups of women who were at a similar stage of pregnancy, talking about parenthood specifically. This was often enough to provide commonalities that transcended other differences between the women, in age for example. Indeed, it is likely that much of the appreciation of the group as a medium of delivery is generalizable to other social interventions aside from parenting, if commonalities in experiences were also present generating support.

A key limitation of the analysis is that the interviews were conducted with women who were willing to be interviewed. They tended to have attended most or all of the sessions, and perhaps their agreement to take part in one or more interviews following completion of the intervention indicated a positive affinity towards the intervention and/or the study and, perhaps, a relatively positive group experience. However, the additional health and social care needs of the women who were interviewed reflected the characteristics of the wider THRIVE study population.

What can this analysis tell us about understanding how group-based parenting interventions might be effective, particularly with vulnerable mothers? The group context did facilitate the women’s engagement with the programmes generally, and the content of the sessions. More than that, the analysis suggests that, as outlined by Schelbe et al. (2018), there is value in the connection the groups offered. Feeling accepted and supported could potentially lead to more positive feelings about parenting, greater confidence and fewer anxieties around motherhood. The group context might be the “active ingredient” which, if there in large enough quantity, may be critical to the effectiveness of the intervention (Craig et al., 2008; Wells et al., 2012). However, it is likely that longer interventions or, more pragmatically, more enduring support beyond the short period of intervention delivery is required. A promising example includes small groups of women with similar health problems keeping in touch beyond the birth of their babies, supporting each other based on what they learnt about each other in the groups (see Sheffield Morris et al., 2017), as well as what they learnt from the groups. As such, social support based on parenting is created beyond the intervention period (see Scott et al., 2001; Strange et al., 2016). However, this process was reported by only a small number of the women. It is likely that the short duration of the interventions, patchy attendance, and the reticence on the part of the facilitators to encourage the women to share the kind of personal information about themselves that would make future contact easy, impeded the formation of enduring bonds between the participants. No data was collected on how many of the women went on to join other groups, as a result of having attended MB or ETPB. This may be a promising outcome of attendance that goes beyond the delivery of the programme itself. The women attending the interventions largely experienced the groups as supportive, and few negative aspects around this support were reported. More group sessions, less patchy attendance, and more encouragement from facilitators for the women to keep in touch, and to join other community parent-child groups are likely to have increased feelings of support and connectedness further. This may, in turn, have boosted the interventions’ effectiveness. Parenting is critically important for child health and well-being (Thompson, 2016); support for mothers is beneficial, particularly for those who have, experienced adversity. Research studies should continue to elucidate how group-based parenting interventions can foster support, and to explore how this might bolster long-term positive effects for parents and children.

## Figures and Tables

**Table 1:** Mellow Bumps antenatal session content.

Session	Content
Pre	Meet and Greet: facilitator(s) visit participant at home
1	Put participants at ease; establish safe environment; dispel false myths about motherhood; normalise reactions to babies if these are not complete positive; introduce capacities of babies; reduce maternal stress
2	Reinforce the acceptance of participants in groups; promote health eating, exercise and behaviours; further explore capacities of babies and how parents can respond; reduce maternal stress
3	Address past interferences or support for being a parent; understand brain development in utero and in the neonate; reduce maternal stress
4	Allow participants the opportunities to explore issues from their past which may impact on their future’explore and discuss babies’communication; promote sharing between participants and supporters; reduce maternal stress
5	Outing or activity
6	Plan future support for mother and baby; make an enduring link in mins of participants; evaluate group; end group positively; reduce maternal stress
7	Optional: session involving fathers/partners

Source: Buston et al. (2019).

**Table 2:** Enhanced Triple P for baby antenatal session content.

Session	Content
1	Positive parenting
2	Responding to your baby: education about infant development
3	Survival skills: raising awareness of the connection between feelings and behaviours and how these impact parents’interactions with their baby
4	Partner support: development of skills to improve partner relationships and manage the stresses of parenting

Source: O’Brien et al. (2019).

**Table 3:** Post-intervention questionnaire responses for mellow bumps and enhanced Triple P for baby.

	Mellow bumps					Enhanced Triple P for baby					
	N = 51					N = 63					
	n (%)					n (%)					
Attending the groups has:	Strongly agree	Agree	Unsure	Disagree	Strongly disagree	Strongly agree	Agree	Unsure	Disagree	Strongly disagree	Statistical significance^a^ p
Has helped me feel better about myself	18 (35)	24 (47)	5 (10)	4 (8)	0 (0)	24 (38)	31 (49)	8 (13)	0 (0)	0 (0)	>.05
Has helped me to accept myself^b^	16 (31)	23 (45)	6 (12)	5 (10)	0 (0)	18 (29)	32 (51)	12 (19)	1 (2)	0 (0)	>.05
Has helped me to become aware of how I am feeling	18 (35)	22 (43)	8 (16)	3 (6)	0 (0)	23 (37)	34 (54)	5 (8)	1 (2)	0 (0)	>.05
Has helped me feel more confident in talking to others^c^	19 (37)	24 (47)	5 (10)	3 (6)	0 (0)	21 (33)	25 (40)	12 (19)	3 (5)	1 (2)	>.05
Has helped me feel more confident about being a mother	22 (43)	21 (41)	5 (10)	3 (6)	0 (0)	28 (44)	31 (49)	3 (5)	0 (0)	1 (2)	>.05
Has helped me feel less anxious about being a mother	18 (35)	16 (31)	13 (35)	4 (8)	0 (0)	24 (38)	30 (48)	7 (11)	2 (3)	0 (0)	>.05
Has given me tips on how to manage stress	18 (35)	26 (51)	6 (12)	1 (2)	0 (0)	25 (40)	36 (57)	1 (2)	1 (2)	0 (0)	>.05
Gave me practical tips on how to care for a new baby	19 (37)	21 (41)	6 (12)	4 (8)	1 (2)	35 (56)	26 (41)	2 (3)	0 (0)	0 (0)	.008
Gave me more understanding of what a baby needs	20 (39)	23 (45)	5 (10)	2 (4)	1 (2)	37 (59)	22 (35)	3 (5)	1 (2)	0 (0)	.024
Has helped me be aware of how I should act with my baby	21 (41)	23 (45)	4 (8)	3 (6)	0 (0)	31 (49)	29 (46)	1 (2)	2 (3)	0 (0)	>.05

a Mann-Whitney.

b 1 missing response for MB.

c 1 missing response for ETPB.
